# Spontaneous Rupture of Hepatocellular Carcinoma: A Rare Cause of Delayed Hemorrhage in Trauma

**DOI:** 10.7759/cureus.85780

**Published:** 2025-06-11

**Authors:** Ryan Moore, Onyinye Nnamdi-Nwosu, Khafra S Garcia Henry

**Affiliations:** 1 General Surgery, University of Central Florida College of Medicine, Ocala, USA; 2 Trauma and Acute Care Surgery, University of Central Florida College of Medicine, Ocala, USA

**Keywords:** case report, embolization, fast exam, hemorrhagic shock, hepatocellular carcinoma, pringle maneuver, trauma

## Abstract

Hepatocellular carcinoma (HCC) accounts for a portion of primary liver cancers worldwide, with hepatitis B virus (HBV) as a major risk factor. Spontaneous rupture of HCC is a rare but life-threatening event. It can be triggered by minor trauma or increased intra-abdominal pressure, necessitating a high index of suspicion in at-risk patients.

A 74-year-old male with HBV presented as a level 2 trauma after a ground-level fall without obvious injuries. Initial evaluation revealed a negative extended focused assessment with sonography in trauma (eFAST). However, the patient subsequently developed hypotension unresponsive to crystalloids and worsening abdominal discomfort. Massive transfusion protocol was initiated, and a repeat FAST revealed free fluid. Emergency laparotomy identified a ruptured right hepatic mass with significant hemorrhage. Hemorrhage control was achieved via hepatic packing, the Pringle maneuver, and right hepatic artery ligation. Postoperatively, angiography with embolization was performed. On postoperative day three, a bile leak required endoscopic retrograde cholangiopancreatography (ERCP) with stent placement. His recovery was otherwise uneventful, and pathology confirmed poorly differentiated HCC.

This case highlights the need for vigilance in trauma patients with risk factors for occult malignancy. The delayed hemorrhage from an undiagnosed HCC rupture emphasizes the role of serial reassessment, prompt surgical intervention, and adjunctive embolization. Trauma surgeons must recognize atypical hemorrhage presentations in at-risk patients. Timely imaging, multidisciplinary management, and early intervention are crucial to improving survival in spontaneous HCC rupture.

## Introduction

Hepatocellular carcinoma (HCC) accounts for over 75% of primary liver cancers worldwide, with chronic hepatitis B virus (HBV) infection being a leading risk factor, contributing to over 40% of global cases [1-4]. The spontaneous rupture of HCC is a life-threatening event, with mortality rates reported between 32% to 100%, primarily due to exsanguination [4]. Studies indicate that spontaneous rupture occurs in 3% to 15% of HCC patients, often precipitated by minor trauma or increased intra-abdominal pressure. Emergency laparotomy with damage control techniques such as hepatic packing and the Pringle maneuver has been shown to improve survival, while subsequent transarterial embolization (TAE) remains a vital adjunct to definitive hemorrhage control [4-7]. The prompt recognition and multidisciplinary management of these cases are crucial to reducing morbidity and mortality.

## Case presentation

A 74-year-old male with a history of hepatitis B presented as a level 2 trauma after a mechanical ground-level fall with a known head strike and no external signs of trauma. Initially, the patient's evaluation revealed hypertension with a negative extended focused assessment with sonography for trauma (eFAST). On completing the secondary survey, whilst awaiting transport to the CT scanner, he gradually developed abdominal discomfort. Repeat vital signs (15 minutes after arrival) showed hypotension (systolic blood pressure below 70), unresponsive to a 2L crystalloid bolus. Massive transfusion protocol was initiated, and 1g tranexamic acid (TXA) was administered. Increasing abdominal distention was noted on exam, and repeat eFAST revealed free fluid in the upper quadrants. Lab workup showed hemoglobin 9.2, an international normalized ratio (INR) of 1.4, and a normal thromboelastogram (TEG) at admission. 

The patient was taken for damage control laparotomy, where he was noted to have significant hemorrhage from a ruptured right hepatic mass. Initial hemorrhage control was obtained via hepatic packing, Pringle maneuver, and ligation of the right hepatic artery. Temporary abdominal closure was performed, and the patient was taken for angiography with embolization of the proper hepatic artery (as demonstrated in the composite Video 1). After stabilization and hemorrhage control, the patient was transferred to the ICU for close hemodynamic monitoring. Labs postoperatively showed elevated liver function tests (hyperbilirubinemia of 4.7 and transaminitis of 496/298). On postoperative day (POD) two, the patient underwent re-exploration with fascial closure and placement of perihepatic Jackson-Pratt (JP) drains. 

**Video 1:** Composite video including eFAST exam, venous, and arterial phase CT imaging, and interventional radiology angiogram This video includes: (1) Positive eFAST examination showing fluid in the hepatorenal space, (2) venous phase CT showing HCC, (3) arterial phase CT demonstrating right hepatic artery ligation, and (4) interventional radiology angiogram showing evidence of flow through a left segment 2 proper hepatic artery branch with a common bifurcation for segments 3 and IVa/IVb with subsequent gelfoam embolization of the proper hepatic artery. eFAST: Extended focused assessment with sonography in trauma, HCC: Hepatocellular carcinoma

Postoperative day three revealed a bilious drain output, for which gastroenterology was consulted for endoscopic retrograde cholangiopancreatography (ERCP). On POD four, the ERCP revealed a stricture at the porta hepatis with proximal dilation of the right and left intrahepatic ducts. This stricture was stented, and a sphincterotomy was performed. The remaining hospital course was notable for decreasing drain output with a change in fluid character to serosanguinous output, and normalization of bilirubin levels. 

However, it was then complicated on POD nine with the development of deep vein thrombosis (DVT). Due to the patient's high risk for bleeding, the decision for an inferior vena cava filter (IVCF) over anticoagulation was made in conjunction with the vascular surgery team. The IVCF placement was performed on POD 10. The patient was subsequently discharged on POD 12 to an inpatient rehabilitation center, where his stay was unremarkable, and he was discharged after 15 days. 

The day after being discharged from the rehabilitation center, he presented at the emergency department (ED) with fevers and chills. A CT of his abdomen showed a large hepatic abscess. He was then admitted for IV antibiotics, percutaneous drainage of the abscess, and infectious disease evaluation. After gradual improvement and de-escalation of antibiotics, the patient was discharged on hospital day seven with a four-week course of outpatient IV ceftriaxone, a one-week course of oral metronidazole, and a hepatology referral. 

Ten days after discharge, the patient called the surgery clinic complaining of a low-grade fever and an elevated heart rate, for which he was instructed to return to the ED. Upon arrival, however, he was afebrile with vital signs within normal limits, and his percutaneous drain was functioning appropriately. He was discharged from the ED. 

## Discussion

This case underscores the necessity of maintaining a broad differential diagnosis in trauma patients, as delayed hemorrhagic manifestations may indicate occult pathology. The presence of an undiagnosed hepatic malignancy contributed in this case to hemorrhage following minor trauma. This highlights the critical role of serial reassessment in trauma patients, prompt surgical intervention, and adjunctive interventional radiological procedures in hemorrhage management. Trauma surgeons must recognize that atypical presentations of hemorrhage, especially in patients with risk factors such as hepatitis B, warrant heightened suspicion for underlying pathology. In HCC rupture, early recognition and timely intervention, including damage control surgery and adjunctive embolization, are critical to improving survival rates [4].

## Conclusions

In trauma patients, the full extent of injuries cannot be assumed based solely on the mechanism of injury or initial stability. Trauma surgeons must recognize atypical hemorrhage presentations in at-risk patients. Clinical reassessment, timely imaging, early intervention, and multidisciplinary management are paramount to identifying and addressing occult injuries. Persistent vigilance contributes to improving patient outcomes in this population.
